# Bryophytes as Strong Aluminum Accumulators in Acidic Soils: Cell-Wall Binding and Physiological Tolerance Mechanisms

**DOI:** 10.3390/plants15121877

**Published:** 2026-06-17

**Authors:** Roghieh Hajiboland, Aiuob Moradi, Hedieh Majmoueh-Koub, Roser Tolrà, Ana Paravinja, Milos Stanojevic, Miroslav Nikolic, Charlotte Poschenrieder

**Affiliations:** 1 Department of Plant, Cell and Molecular Biology, University of Tabriz, Tabriz 51666-16471, Iran; hedyeh.majkoob1402@ms.tabrizu.ac.ir; 2 Forests and Rangelands Research Department, Guilan Agricultural and Education Center, Agricultural Research, Education and Extension Organization (AREEO), Rasht 41635-3394, Iran; aiuobmoradi50@gmail.com; 3 Plant Physiology Laboratory, Bioscience Faculty, Universidad Autónoma de Barcelona, 08193 Bellaterra, Spain; roser.tolra@uab.cat; 4 Institute for Multidisciplinary Research, National Institute of the Republic of Serbia, University of Belgrade, 11030 Belgrade, Serbia; ana.paravinja@imsi.bg.ac.rs (A.P.); milos.stanojevic@imsi.bg.ac.rs (M.S.); mnikolic@imsi.bg.ac.rs (M.N.); 5 Faculty of Sciences and Mathematics, University of Pristina, 38220 Kosovska Mitrovica, Serbia

**Keywords:** aluminum accumulation, aluminum sequestration, callose, carboxylic acids, cell-wall binding, liverworts, mosses, pectin

## Abstract

Bryophytes are key components of acid–soil ecosystems; however, their capacity for aluminum (Al) accumulation and tolerance remains poorly understood. In this study, bryophytes and a limited number of pteridophyte and lichen species were collected from acidic soils of tea plantations and adjacent forest stands in the Caspian region of northern Iran and analyzed. Nearly all bryophyte specimens exhibited Al concentrations above the critical accumulation threshold (1000 µg g^−1^ DW), with some reaching values exceeding 28,000 µg g^−1^ DW, confirming their strong accumulation capacity. After Al, iron was the most abundantly accumulated metal (1430–22,800 µg g^−1^ DW), followed by manganese (100–3100 µg g^−1^ DW). The sampled lichen species accumulated Al at concentrations between 1063 and 9154 µg g^−1^ DW, while Al levels in the aerial parts of pteridophytes rarely exceeded the critical threshold; when they did, accumulation occurred predominantly in old and fertile fronds rather than sterile ones. Three field-collected bryophyte species—*Barbula unguiculata*, *Palamocladium euchloron*, and *Hypnum cupressiforme*—were acclimated to laboratory conditions and treated with two Al levels (without or with 150 µM Al, pH 4.0) for 12 weeks. The leafy shoots were analyzed for their antioxidant response, osmolyte accumulation, phenolic metabolism, callose deposition, and carboxylic-acid profile. Histochemical analyses revealed predominant localization of Al in cell walls, associated with enrichment of pectin and uronic acids. These responses were most pronounced in *H. cupressiforme*, followed by *P. euchloron*, and least evident in *B. unguiculata*. Elevated levels of intracellular detoxification compounds—phenolics, flavonoids, and carboxylic acids (tartaric, oxalic, malic, and citric acids)—were detected, again with species-specific differences. Overall, the results reveal that bryophytes employ multiple physiological strategies to tolerate Al toxicity, with substantial interspecific variation. These findings emphasize their ecological significance and provide a foundation for future research on the physiological and evolutionary mechanisms underlying Al tolerance and accumulation in early land plants.

## 1. Introduction

Acidic soils (pH in water < 5.5) are characterized by high proton activity and distinct mineralogical and chemical processes that strongly influence the solubility and bioavailability of metals [1,2]. Under low-pH conditions, enhanced protonation promotes the dissolution of primary and secondary minerals, particularly aluminosilicates and aluminum oxides, leading to the release of aluminum (Al) into the soil solution. Consequently, Al occurs predominantly in soluble and exchangeable forms under acidic conditions, such as Al^3+^ and hydroxy–aluminum complexes (e.g., [Al(H_2_O)_4_(OH)_2_]^+^ and [Al(H_2_O)_5_(OH)]^2+^), which are readily available for plant uptake [1,2]. Acidic conditions also increase the mobility of other metals, including iron (Fe), manganese (Mn), and various trace elements, through similar dissolution and desorption processes. Collectively, these chemical characteristics make acidic soil environments with elevated metal bioavailability, where rhizotoxic Al^3+^ is a major factor influencing plant nutrition, growth, and species distribution [1].

Acidic soils support a distinct flora composed largely of species that have evolved physiological and structural adaptations to tolerate low pH, elevated Al^3+^ activity, and associated nutrient imbalances [3]. Globally, vegetation on acidic substrates predominantly comprises acidophilic and calcifuge species that are poorly competitive in or entirely excluded from neutral to calcareous soils. Common vascular plant families characteristic of acidic soils include Ericaceae, Rubiaceae, Poaceae, Cyperaceae, Juncaceae, and Melastomataceae [4,5,6]. A recent survey of acidic-soil vegetation in the tea gardens and adjacent areas of northern Iran documented numerous annual and perennial herbaceous species, including 36 Al-accumulating taxa belonging to 23 families [7]. The ecological success of these species has been primarily attributed to two key adaptive strategies: (1) efficient exclusion of Al^3+^ from the roots and (2) effective internal sequestration of Al within plant tissues [3,8]. Consequently, acidophilic flora are valuable model systems for investigating plant–soil interactions, Al toxicity tolerance mechanisms, and metal accumulation under stressful edaphic conditions.

Bryophytes are ubiquitous and important components of acid–soil ecosystems [9] and may also contribute to soil acidification processes [10]. However, bryophytes and other non-vascular plants have received comparatively little attention in studies specifically addressing Al uptake and accumulation. Most existing research on bryophyte–metal interactions has focused on heavy metals such as copper (Cu), zinc (Zn), lead (Pb), mercury (Hg), and cadmium (Cd), largely in the context of environmental monitoring and pollution assessment [11,12]. Due to their poikilohydric nature, absence of a cuticle and vascular tissues, large surface-to-volume ratio, and ability to absorb ions across the entire gametophyte surface, bryophytes (particularly mosses) are widely used as biomonitors of atmospheric metal deposition and as model organisms in remediation studies [13]. These studies demonstrate the remarkable capacity of bryophytes to accumulate metals through processes such as surface adsorption, ion exchange, particulate entrapment, and intracellular uptake [14].

In vascular plants, Al tolerance and resistance are largely mediated by root-based mechanisms, including enhanced carboxylic acid exudation into the rhizosphere, restricted root-to-shoot transport of Al^3+^, and vacuolar compartmentalization of Al [1,15]. These strategies are inherently dependent on the presence of true roots and vascular tissues, structures that bryophytes lack. Whether physiological and biochemical processes described in seed plants, such as intracellular metal sequestration, organic ligand complexation, or tissue-level compartmentalization, also contribute, directly or indirectly, to Al accumulation and tolerance in bryophytes remains largely unknown.

Northern Iran, particularly the Caspian Sea region and the northern slopes of the Alborz Mountains, where high annual precipitation, dense forest cover, and intense weathering of parent materials have contributed to the widespread development of acidic soils across many forested and montane sites, is an ecologically important area for studying acidic-soil vegetation and metal dynamics [7]. The Hyrcanian forests and associated montane habitats support a rich community of bryophyte flora, including both terricolous species inhabiting forest floors and epiphytic taxa growing on tree trunks and branches [16,17,18]. Despite this ecological significance, systematic studies addressing Al accumulation in bryophytes from northern Iran remain scarce. Incorporating this regional context, therefore, broadens the geographical scope of Al–plant interaction research and provides baseline information from an understudied biodiversity hotspot.

Aluminum accumulation is a relatively uncommon trait among higher plants, occurring in only about 5% of angiosperm species [5]. We hypothesized that Al accumulation is more widespread in bryophytes inhabiting acidic environments than in vascular plants, owing to their lack of true roots and limited capacity for selective ion uptake. We further hypothesized that bryophyte species differ in their capacity for Al accumulation and in the physiological and biochemical mechanisms underlying Al tolerance. To test these hypotheses and address the limited understanding of Al dynamics in non-vascular plants, we evaluated Al accumulation in bryophytes from acidic substrates and examined its relationships with other metals. A second objective was to explore the physiological and biochemical mechanisms potentially involved in Al accumulation and tolerance in bryophytes. By integrating field sampling and metal concentration measurements with biochemical analyses, this study provides a framework for evaluating Al accumulation strategies in bryophytes and contributes to a broader understanding of plant–metal interactions across divergent plant lineages in acidic-soil ecosystems.

## 2. Results

### 2.1. Accumulation of Al in Collected Bryophyte Specimens

All bryophyte specimens exhibited Al concentrations above the critical threshold defining Al accumulator species (1000 µg g^−1^ DW; Table 1) [5]. The highest average Al concentrations, approaching ~28,000 µg g^−1^ DW, were recorded in two liverworts, *Fossombronia caespitiformis* and *Lunularia cruciata*. In contrast, the lowest Al concentrations were observed in the mosses *Neckera complanata* and *Oxyrrhynchium hians*, both of which accumulated 1600–1900 µg g^−1^ DW (Table 1).

*Palamocladium euchloron* was the most abundant and widespread species in the study area, occurring in tea gardens, adjacent habitats, and forest sites. In samples separated into young and old shoots, Al accumulation was significantly higher in the old shoots than in the young shoots (Table S1). Moreover, *P. euchloron* specimens collected from tea gardens exhibited significantly greater whole-shoot Al concentrations than those collected from forest habitats (Table S1).

The effect of substrate on Al accumulation was evaluated in *P. euchloron*, which occurred on both stone surfaces and tree bark, and in *Amblystegium serpens*, another common species found exclusively in tea gardens which grows on soil, stone, and tree bark. Statistical analyses revealed no significant differences in Al concentrations among specimens collected from different substrates in either species (Table S1).

### 2.2. Accumulation of Al in Other Non-Seed Plants Collected

In pteridophytes, Al concentrations in aerial parts showed considerable variability, ranging from <200 to >1000 µg g^−1^ DW depending on the species (Table 1). Analyses of distinct plant organs revealed that the stipes of *Asplenium adiantum-nigrum* and *Dryopteris raddeana*, as well as the fertile fronds of *A. adiantum-nigrum*, contained high Al levels (Table S2) exceeding the accumulation threshold [5]. Higher Al concentrations were predominantly found in rhizomes, reaching 1123 µg g^−1^ DW in *A. adiantum-nigrum* and 4525 µg g^−1^ DW in *Polypodium vulgare* (Table S2). Overall, one-way ANOVA excluding rhizomes indicated that older or reproductive plant parts accumulated significantly more Al than young or sterile plant parts across all four fern species (Table S3).

All four lichen species accumulated Al above the critical threshold. The highest concentration was observed in *Lepraria leuckertiana* (9154 µg g^−1^ DW), followed by *Xanthoria parietina* (4885 µg g^−1^ DW). The other two species accumulated between 1063 and 3541 µg g^−1^ DW (Table 1).

### 2.3. Accumulation of Other Elements in Collected Bryophyte Specimens and Its Correlation with Al

**Figure 1:**
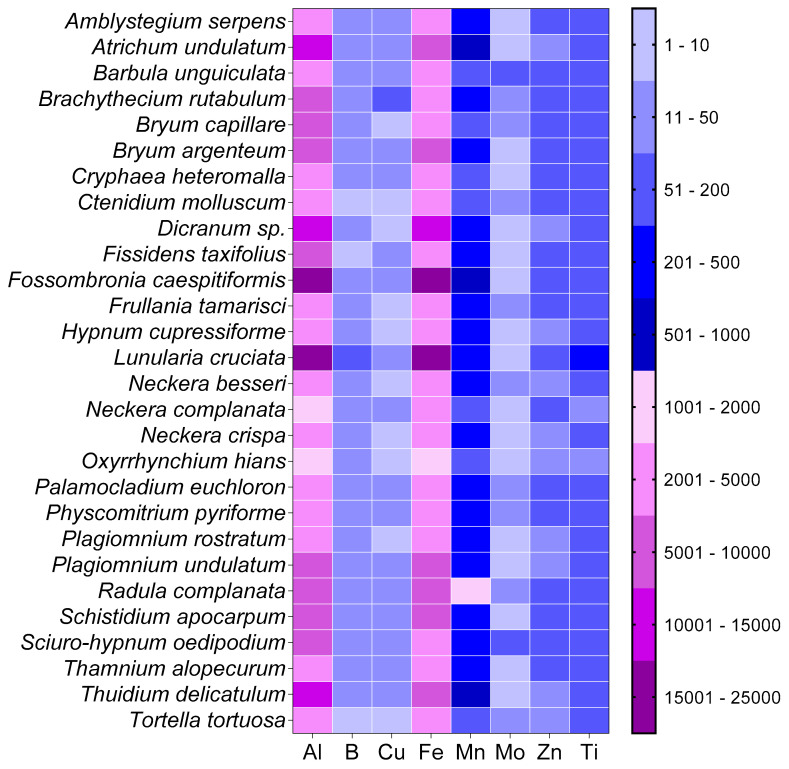
Concentrations (µg g^−1^ DW) of Al, micronutrients (B, Cu, Fe, Mn, Mo, Zn), and Ti in bryophyte species collected from acidic soils in tea gardens and Hyrcanian forests of northern Iran.

Among micronutrients, Fe showed the highest concentrations, comparable to the range observed for Al (1430–22,800 µg g^−1^ DW). Manganese concentrations were lower, generally ranging from 100 to 3100 µg g^−1^ DW (Figure 1). Concentrations of non-nutrient heavy metals typically fell within 1–80 µg g^−1^ DW, except for Cd, which was mostly below 1.0 µg g^−1^ DW (Figure S1).

**Figure 2:**
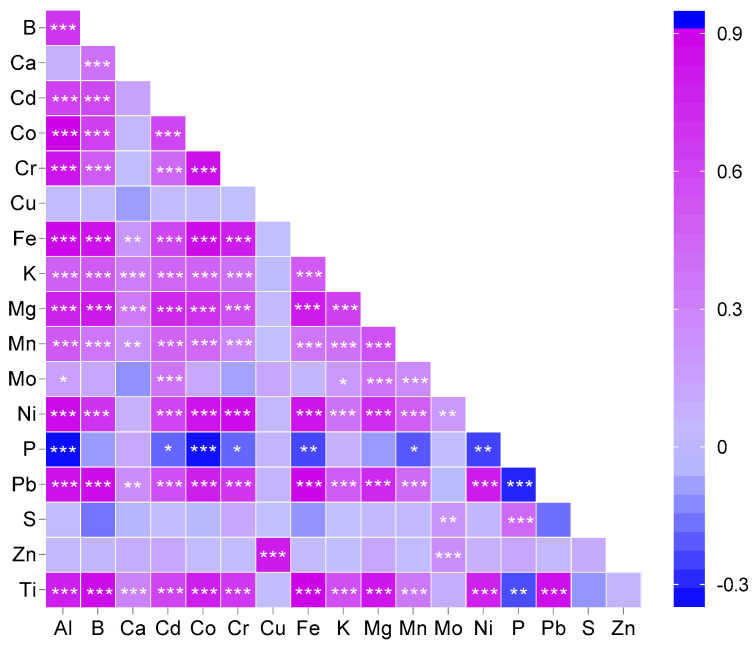
Correlations between the concentrations of different elements in bryophyte species collected from acidic soils in tea gardens and Hyrcanian forests of northern Iran. Entries represent Pearson correlation coefficients. * *p* < 0.05; ** *p* < 0.01, *** *p* < 0.001.

Significant positive correlations were observed between Al and the macronutrients potassium (K) and magnesium (Mg); the micronutrients boron (B), Fe, Mn, and nickel (Ni); and the non-nutrient heavy metals Cd, cobalt (Co), chromium (Cr), and Pb. In contrast, a significant negative correlation was found between Al and phosphorus (P) concentrations (Figure 2). Titanium (Ti), which was included to assess potential contributions from dust deposition or soil contamination, showed concentrations of 40–300 µg g^−1^ DW and, similar to the other heavy metals, exhibited a positive correlation with Al (Figure 2).

### 2.4. Aluminum Concentration, Photosynthetic Pigments, Osmolytes, and Callose in the Leafy Shoots of Field-Collected, Laboratory-Acclimated Bryophyte Species

Three moss species, *Barbula unguiculata*, *Palamocladium euchloron*, and *Hypnum cupressiforme*, were collected from the field, acclimated to laboratory conditions, and subsequently exposed to two Al treatments (−Al and +Al, 150 µM Al). Following 12 weeks of cultivation under these conditions, all three moss species accumulated significantly higher concentrations of Al in their leafy shoots compared with the −Al controls (Table 2).

A significant reduction in chlorophyll concentrations was observed following Al exposure across all species. Specifically, both chlorophyll *a* and chlorophyll *b* decreased significantly in *B. unguiculata*, whereas the Al treatment reduced only chlorophyll *a* in *P. euchloron* and only chlorophyll *b* in *H. cupressiforme*. Furthermore, carotenoid levels declined significantly in all three species following Al exposure (Table 2).

Soluble carbohydrate levels showed a significant increase across all species examined. However, the response of starch to Al treatment varied among species: *P. euchloron* exhibited a decrease in starch content, *H. cupressiforme* showed an increase, and *B. unguiculata* remained unchanged. Proline concentrations decreased in *B. unguiculata* and *P. euchloron* under Al treatment, but this reduction remained non-significant in *H. cupressiforme*. Callose concentrations tended to be lower in Al-exposed mosses, but a statistically significant reduction was detected only in *P. euchloron* (Table 2).

### 2.5. Accumulation and Localization of Al in Field-Collected, Laboratory-Acclimated Bryophyte Species

**Figure 3:**
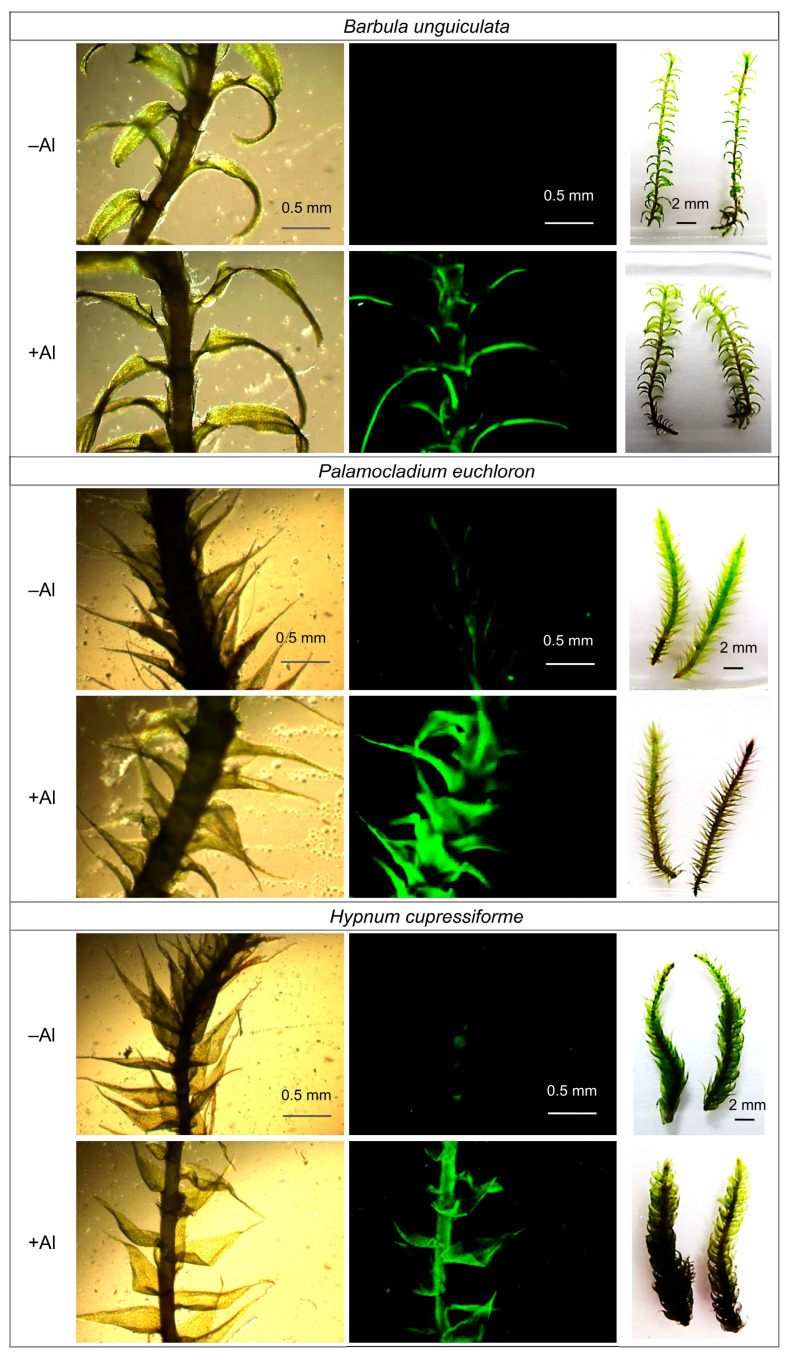
Detection of Al using morin (middle panels) and hematoxylin (right panels) in three bryophyte species cultivated without Al (–Al) or with 150 µM Al (+Al, as AlCl_3_) at pH 4.0 for 12 weeks under controlled environmental conditions. Corresponding light microscopy images are shown in the left panels.

Morin staining of laboratory-acclimated moss species revealed Al deposition throughout the leafy shoots of +Al mosses. Fluorescence intensity was the highest in *P. euchloron*, followed by *H. cupressiforme*, and the lowest in *B. unguiculata* (Figure 3). Hematoxylin staining confirmed these results: Al deposition was particularly prominent in the lower portions of leafy shoots, and *B. unguiculata* consistently showed weaker staining than the other two species (Figure 3). It is noteworthy that the cultivated bryophytes showed no specific symptoms when treated with Al. Instead, they developed leafy shoots more rapidly, with denser mats relative to the treatments lacking Al (Figure S2).

### 2.6. Antioxidant Defense Responses of Al-Treated Mosses

**Figure 4:**
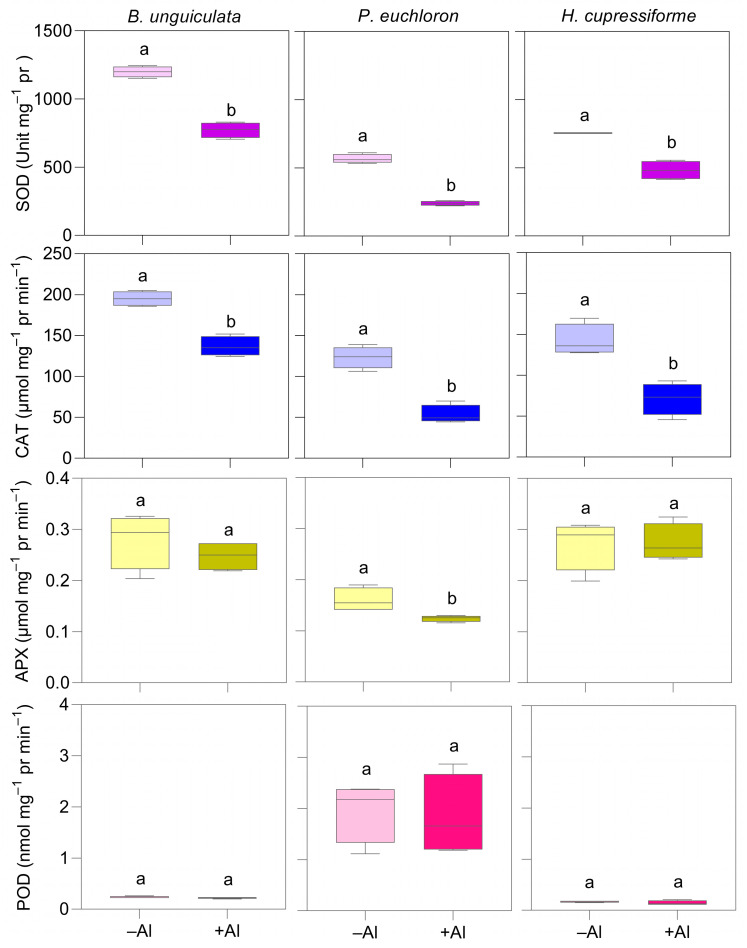
Activities of superoxide dismutase (SOD), catalase (CAT), ascorbate peroxidase (APX), and peroxidase (POD) in leafy shoots of three bryophyte species cultivated without Al (–Al) or with 150 µM Al (+Al, as AlCl_3_) at pH 4.0 for 12 weeks under controlled environmental conditions. Bars indicated by the same letter are not significantly different at *p* < 0.05.

Superoxide dismutase (SOD) and catalase (CAT) activity declined significantly in all species under Al exposure. Ascorbate peroxidase (APX) activity remained unchanged in *B. unguiculata* and *H. cupressiforme* but decreased significantly in *P. euchloron*. Peroxidase (POD) activity was unaffected by Al treatment in all species, although *P. euchloron* exhibited a notably higher basal POD activity—approximately 15-fold greater—than the other species (Figure 4).

**Figure 5:**
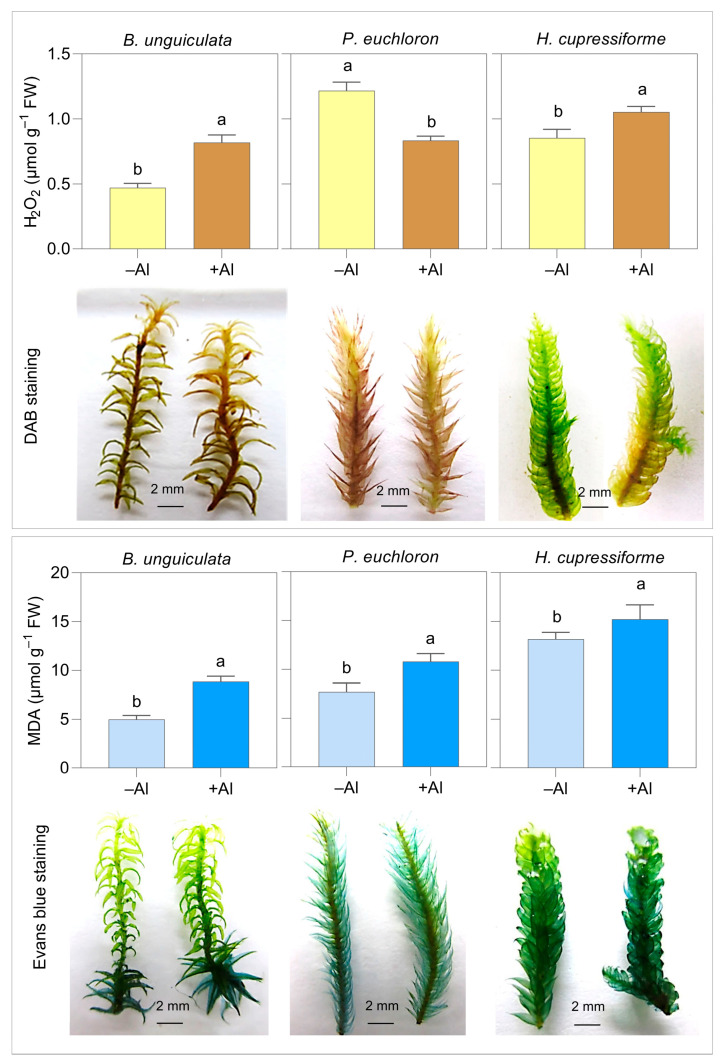
Concentration of hydrogen peroxide (H_2_O_2_) and its histochemical detection using DAB staining, malondialdehyde (MDA) concentration, and detection of cell damage using Evans blue staining in leafy shoots of three bryophyte species cultivated without Al (–Al) or with 150 µM Al (+Al, as AlCl_3_) at pH 4.0 for 12 weeks under controlled environmental conditions. Bars indicated by the same letter are not significantly different at *p* < 0.05.

Hydrogen peroxide (H_2_O_2_) concentrations increased significantly in Al-exposed *B. unguiculata* and *H. cupressiforme* but decreased in *P. euchloron*. DAB staining corroborated these patterns (Figure 5). In contrast, malondialdehyde (MDA) levels increased significantly in all three species under Al treatment, with no substantial interspecific differences. According to the results of Evans blue staining, membrane damage was concentrated in the lower portions of leafy shoots in *B. unguiculata* and *H. cupressiforme*, and injury was more pronounced under Al exposure. In *P. euchloron*, staining was distributed more uniformly across the leafy shoots (Figure 5).

### 2.7. Activity of Phenolic-Metabolizing Enzymes and Concentrations of Al-Chelating Compounds in Al-Treated Mosses

**Figure 6:**
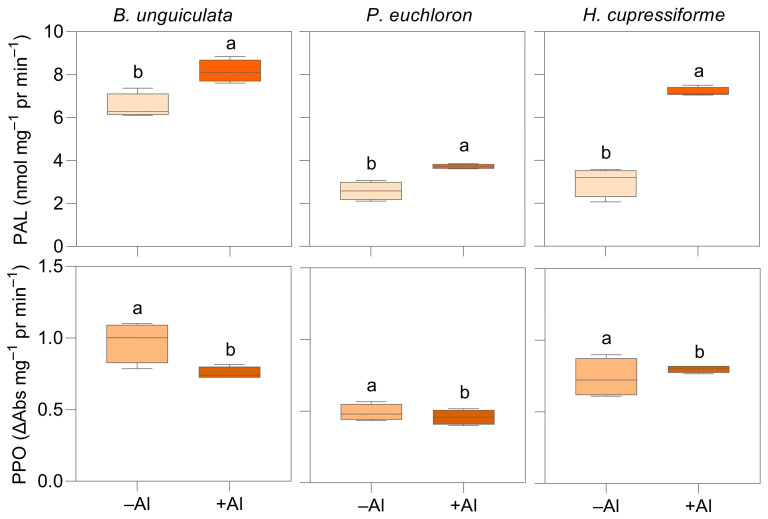
Activities of phenylalanine ammonia-lyase (PAL) and polyphenol oxidase (PPO) in leafy shoots of three bryophyte species cultivated without Al (–Al) or with 150 µM Al (+Al, as AlCl_3_) at pH 4.0 for 12 weeks under controlled environmental conditions. Bars indicated by the same letter are not significantly different at *p* < 0.05.

**Figure 7:**
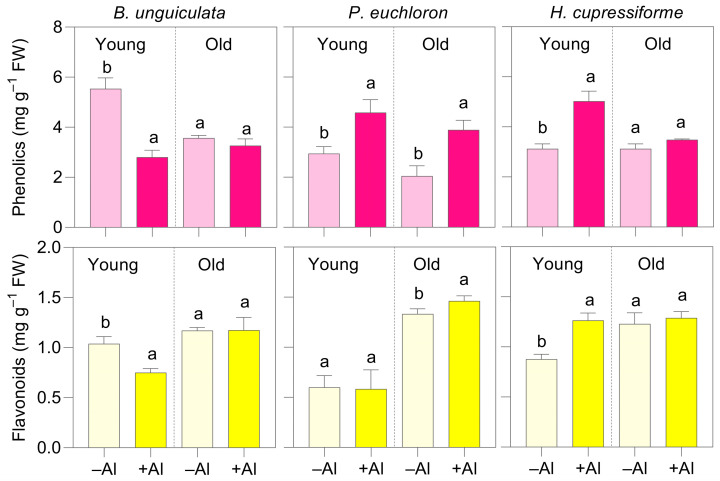
Concentrations of total phenolics and flavonoids in young and old shoots of three bryophyte species cultivated without Al (–Al) or with 150 µM Al (+Al, as AlCl_3_) at pH 4.0 for 12 weeks under controlled environmental conditions. Bars within each shoot age class indicated by the same letter are not significantly different at *p* < 0.05.

Following Al exposure, phenylalanine ammonia-lyase (PAL) activity increased and polyphenol oxidase (PPO) activity decreased in all three species (Figure 6). In *B. unguiculata*, phenolics and flavonoids decreased under Al in the young shoots but remained unchanged in the older ones. In *P. euchloron*, phenolics increased in both young and old shoots, while flavonoids increased only in the old shoots. In *H. cupressiforme*, both phenolics and flavonoids increased in the young shoots but were unaffected in the older ones (Figure 7).

**Figure 8:**
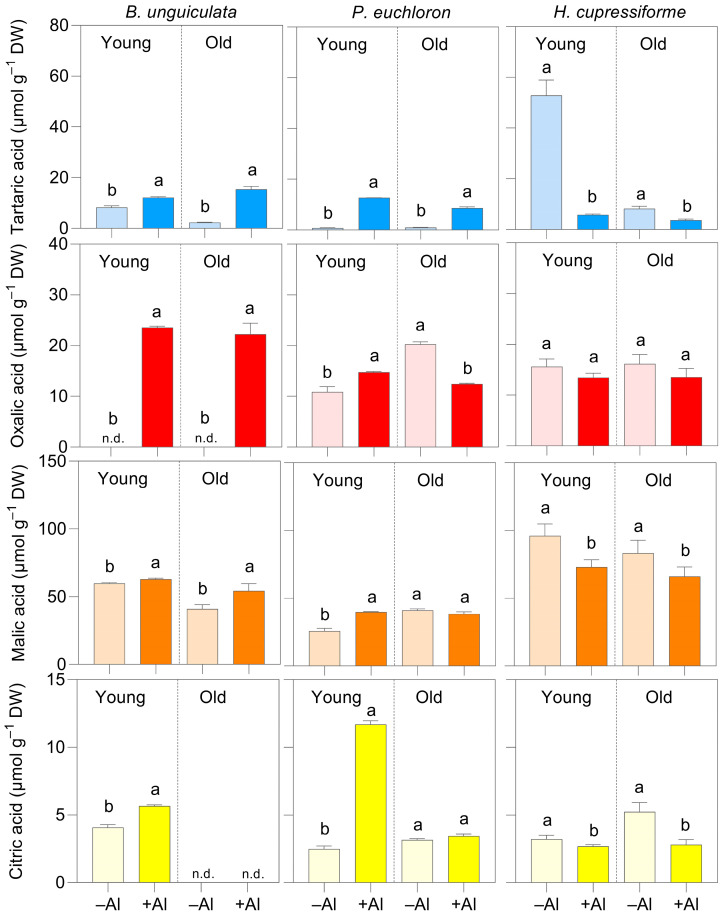
Concentrations of five carboxylic acids (tartaric, oxalic, malic, and citric acids) in young and old shoots of three bryophyte species cultivated without Al (–Al) or with 150 µM Al (+Al, as AlCl_3_) at pH 4.0 for 12 weeks under controlled environmental conditions. Bars within each shoot age class indicated by the same letter are not significantly different at *p* < 0.05.

In *B. unguiculata*, tartaric, oxalic, malic, and citric acids increased significantly in both young and old shoots. However, citric acid was not detected in the old shoots. In *P. euchloron*, all carboxylic acids increased in the young shoots. In the old shoots, only tartaric acid increased; oxalic acid decreased, and malic and citric acids remained unchanged (Figure 8). In *H. cupressiforme*, concentrations of all measured carboxylic acids decreased consistently in both young and old shoots, except for a non-significant reduction in malic acid content in the old shoots (Figure 8).

### 2.8. C_2_ Metabolism Intermediates and Formic Acid in Al-Treated Mosses

Glyoxylic acid increased in *B. unguiculata* and *P. euchloron* but decreased in *H. cupressiforme*. Similarly, glycolic acid increased in *B. unguiculata*, whereas in the other two species, the response differed between leafy shoots of different age classes, decreasing in young shoots and increasing in old shoots (Table S4). The concentration of formic acid increased in both young and old shoots of all three species in response to Al exposure; this effect was most pronounced in *H. cupressiforme* (Table S4).

### 2.9. FTIR Analysis and Cell-Wall Characteristics in Al-Treated Mosses

**Figure 9:**
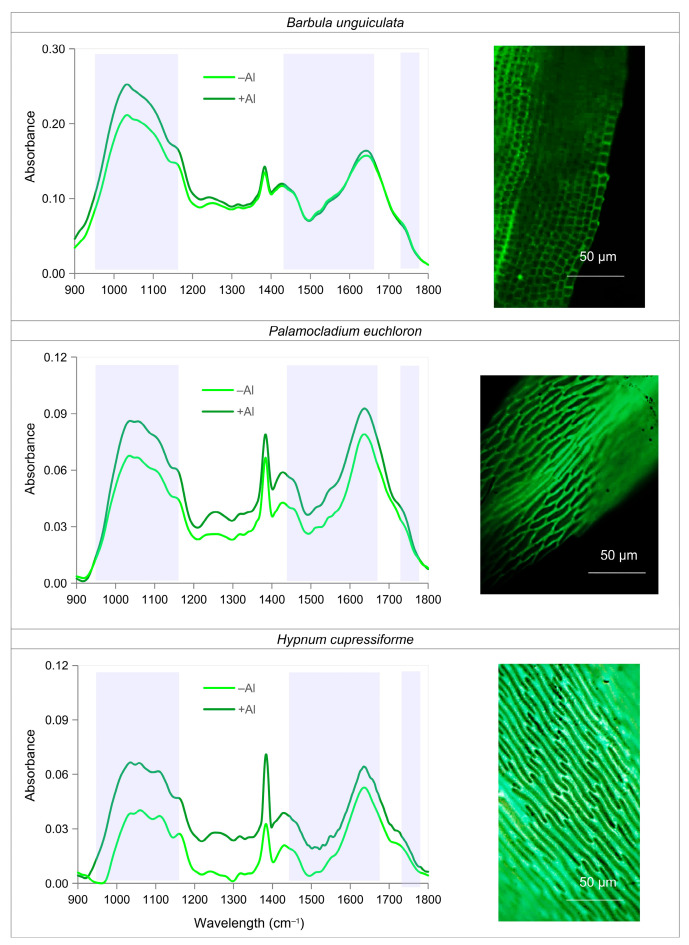
Fourier transform infrared (FTIR) spectra (left panels) of leafy shoots and morin staining of leaves (right panels) in three bryophyte species cultivated without Al (–Al) or with 150 µM Al (+Al, as AlCl_3_) at pH 4.0 for 12 weeks under controlled environmental conditions.

Fourier transform infrared (FTIR) spectra in the regions indicative of esterified uronic acids (1740 cm^−1^), non-esterified uronic acids (1420–1630 cm^−1^), and pectins (960 and 1140 cm^−1^) showed species-specific responses to Al. Enhanced absorption across the 900–1800 cm^−1^ region was most pronounced in *H. cupressiforme*, followed by *P. euchloron*. In *B. unguiculata*, Al-treated mosses differed only in the pectin-associated bands (960 and 1140 cm^−1^), with little or no change in the uronic acid-related regions (Figure 9). Morin-stained leaf cells revealed marked interspecific differences in cell-wall-associated Al: *H. cupressiforme* showed the thickest and most intense cell-wall staining, followed by *P. euchloron*, whereas *B. unguiculata* exhibited the weakest staining (Figure 9).

## 3. Discussion

Ecological, geobotanical, and physiological research on acidic soils has historically focused on seed plants, leading to a substantial bias in our understanding of plant–Al interactions [1,4,5,6]. As a result, existing models of Al tolerance, exclusion, and sequestration were derived almost entirely from studies on higher plants, whereas comparable mechanisms in non-vascular taxa remain largely unexplored. Despite being widespread and frequently dominant in acidic habitats, bryophytes have received comparatively little attention regarding their capacity for Al uptake and accumulation. This study addresses this gap by examining Al accumulation patterns in bryophytes collected from naturally acidic substrates in northern Iran.

The sampled sites in northern Iran are ecologically distinctive acidic soil environments shaped by the unique climatic and edaphic conditions of the Hyrcanian region [7]. The Hyrcanian forest belt constitutes one of Southwest Asia’s primary biodiversity hotspots, characterized by exceptionally rich flora and vegetation [17,18] and designated as a UNESCO World Heritage Site (https://whc.unesco.org/en/list/1584, accessed on 13 May 2026). These acidic soil habitats support plant assemblages, including bryophytes, that are adapted to both regional climate and persistent soil acidity, providing an informative natural system for evaluating Al accumulation in non-vascular plants.

### 3.1. Widespread and Substantial Al Accumulation in Bryophytes

All 28 bryophyte species collected from acidic soils accumulated Al at concentrations exceeding the commonly accepted threshold for Al-accumulator plants (1000 µg g^−1^ DW) [5]. The recorded concentrations (2000–28,000 µg g^−1^ DW) fall within the upper range, and in some cases even exceed those reported for several well-known Al-accumulating seed plant species [4,5,6].

The highest Al concentrations reported for higher plants (e.g., 30,500 µg g^−1^ DW in *Memecylon laurinum*) occur predominantly in tropical woody species [6], where long leaf longevity may contribute substantially to cumulative Al storage. In contrast, the bryophytes analyzed in this study consisted entirely of actively growing green tissues, with gametophyte shoots largely representing recent annual growth. This difference may indirectly indicate a considerably higher capacity for Al uptake in bryophytes compared with woody vascular plants. Consistent with the role of tissue longevity in Al accumulation, a comparison between young and old shoots within a single species (*P. euchloron*) revealed significantly higher Al concentrations in older shoots (Table S1).

Although the highest Al concentrations (about 26,000–28,000 µg g^−1^ DW) occurred in two liverwort species (*Fossombronia caespitosa* and *Lunularia cruciata*), relatively high levels (15,000–24,000 µg g^−1^ DW) were also measured in two mosses (*Sciuro-hypnum oedopodium* and *Dicranum* sp.). In contrast, the liverwort *Frullania tamarisci* showed much lower concentrations (around 4000 µg g^−1^ DW). Overall, these patterns suggest that Al accumulation capacity does not correspond clearly with the broader taxonomic categories of mosses versus liverworts.

There is a close relationship between soil pH and the solubility of Al^3+^ and, consequently, its availability for plant uptake [1,2]. In the present study, using *P. euchloron* as a representative species, specimens collected from tea plantations were found to have accumulated significantly higher concentrations of Al than those collected from adjacent forest sites. This difference is consistent with the lower soil pH observed in tea plantations (pH 3.5–4.0) compared with forest soils (pH 5.5–6.0) (Table S5). The lower pH of tea plantation soils is likely attributable to the long-term application of ammonium-based fertilizers, which promote soil acidification and are commonly preferred over nitrate fertilizers in tea cultivation [7].

Previous studies have suggested that metal concentrations in corticolous bryophytes primarily reflect atmospheric deposition, whereas accumulation in terricolous species is more strongly influenced by soil chemistry and substrate-derived inputs [13]. In the present study, however, Al concentrations in *Palamocladium euchloron* and *Amblystegium serpens* did not differ significantly among specimens collected from different substrates (Table S1). This suggests that substrate type was not a major determinant of Al accumulation in these species and that Al exposure may have been relatively similar across substrates. Although metal accumulation in bryophytes has been extensively studied across species and habitats [11,12,13,14], direct comparisons between corticolous and terricolous species remain limited. Further studies involving a wider range of species, particularly substrate-generalist taxa, are needed to distinguish the effects of substrate characteristics from those of atmospheric deposition and species-specific accumulation mechanisms.

Analyses of the collected pteridophytes (ferns and horsetails) revealed a much lower capacity for Al accumulation in aerial tissues, with concentrations exceeding the Al-accumulator threshold only in some shoot fractions of *Asplenium adiantum-nigrum*, *Dryopteris raddeana*, and *Equisetum telmateia*. A clear organ-specific and age-dependent pattern of Al accumulation was observed in ferns (Tables S2 and S3), paralleling observations in bryophytes and seed plants, where older tissues tended to accumulate higher Al concentrations, most likely because of longer exposure and gradual metal deposition over time. The strong Al accumulation observed in all four analyzed lichen species supports previous reports that lichens efficiently adsorb airborne and substrate-derived metals through extracellular polysaccharide matrices [19].

### 3.2. Acidic Soil Chemistry Drives Coordinated Metal Accumulation in Bryophytes

A notable finding was the positive correlation between the accumulation of Al and that of several other metals, including micronutrients [Fe, Mn, molybdenum (Mo), and Ni] and non-essential metals (Cd, Co, Cr, Pb, and Ti), whereas no correlation was observed with Cu or Zn. Similar correlations have been reported in Al-accumulating seed plants [7] and are generally attributed to the enhanced solubility of many metal cations under low-pH conditions [1]. However, the stronger and more consistent correlations observed in bryophytes in this study suggest that passive surface sorption to cell walls may play a dominant role in metal co-accumulation in these non-vascular plants. The absence of a correlation between Al and either Cu or Zn likely reflects the strong complexation of these elements with soil organic compounds [20] and their comparatively limited mobilization through proton-induced mineral dissolution.

Among macronutrients, calcium (Ca) showed no significant correlation with Al accumulation, whereas K and Mg were positively correlated with Al. Under acidic conditions, Ca is generally more susceptible to leaching because it is weakly retained in soil exchange sites and is readily displaced by H^+^ and Al^3+^ ions [21]. As a result, its availability may be governed primarily by local leaching intensity rather than the processes controlling Al mobilization, which could explain the absence of a consistent relationship between Ca and Al concentrations. In contrast, the positive correlations of Mg and K with Al may reflect their concurrent release during the weathering of soil minerals under acidic conditions. Increasing soil acidity enhances mineral dissolution and the mobilization of Al while simultaneously releasing Mg and K from mineral matrices [22]. Consequently, bryophytes growing in soils with greater Al mobilization may also accumulate higher concentrations of Mg and K, resulting in the observed positive relationships among these elements in bryophyte tissues.

The significant negative correlation between Al and P is consistent with the well-established antagonistic interaction between Al^3+^ and phosphate under acidic conditions [23]. Increased Al availability promotes the formation of insoluble Al–phosphate complexes in the substrate, thereby reducing P availability for plant uptake and contributing to the inverse relationship observed in bryophyte tissues.

Although Mo availability is typically low in acidic soils due to strong adsorption of the molybdate anion (MoO_4_^2–^) onto Fe and Al oxides [24], Mo concentrations in bryophyte tissues were positively correlated with Al. A similar pattern has been reported in Al-accumulating seed plants but not in Al excluders [7]. This association may reflect a coordinated physiological adaptation in Al-accumulating species—an interpretation that warrants further investigation in bryophytes and other native acidophilic flora.

### 3.3. Oxidative Stress and Osmotic Adjustment Responses to Al Exposure in Bryophytes

Exposure of plants to Al^3+^ induces excessive generation of reactive oxygen species (ROS) and disrupts cellular redox homeostasis, causing lipid peroxidation, impairment of enzymatic activity, and pigment degradation [25]. In our study, antioxidant enzyme activity was rarely enhanced under Al treatment. Instead, SOD and CAT activity generally decreased, accompanied by H_2_O_2_ accumulation (except in *P. euchloron*) and increased lipid peroxidation, as indicated by elevated MDA levels and stronger Evans blue staining across all three species. These patterns suggest that boosting antioxidant defenses is not a primary stress mitigation strategy in these bryophytes. It is noteworthy that similar reductions in antioxidant enzyme activity were also observed after short-term (4-week) Al exposure (Table S6), confirming that the suppression of enzymatic antioxidant systems is consistent across exposure durations.

The involvement of compatible solutes in metal stress tolerance is well documented in higher plants [26], although evidence in bryophytes remains limited. In our work, soluble carbohydrates increased in all three species under Al exposure, which may contribute to osmotic adjustment and additional protective functions such as protein stabilization and ROS buffering [27]. This increase was accompanied by starch depletion in *P. euchloron*, whereas *H. cupressiforme* exhibited increases in both soluble carbohydrates and starch, suggesting species-specific differences in the metabolic sources of soluble sugars.

Proline is a key cellular protectant involved in osmotic adjustment, membrane stabilization, ROS scavenging, and the preservation of protein structure and function [28]. Proline accumulation has been reported in mosses such as *Atrichum undulatum* and in the liverwort *Marchantia polymorpha* in response to drought stress [29,30]. In higher plants, numerous studies have shown that proline accumulation under metal stress, including Al exposure, is associated with upregulation of its biosynthetic pathway and can contribute to genotype-specific tolerance [31]. However, evidence for proline-mediated metal-stress responses in bryophytes is scarce. In our study, proline levels decreased under Al exposure except in *H. cupressiforme*, where levels remained unchanged. Studies on three bryophyte species exposed to Cd stress showed species-dependent responses in proline levels, with both increases and decreases reported [32], highlighting that proline-related responses in bryophytes are highly species-specific.

### 3.4. Phenolic Compounds Indicate Metabolic Adjustments and Detoxification Responses

Low-molecular-weight metal-chelating compounds, such as phenolics and carboxylic acids, contribute to Al^3+^ chelation and detoxification in higher plants, acting both externally in the rhizosphere and internally within cells [33,34]. In bryophytes, however, root-mediated exudation of chelators is unlikely to be a major strategy because they lack well-developed roots and primarily absorb elements across their entire surface rather than through root tissues alone. Consequently, internal Al detoxification through the chelation of Al^3+^ by phenolic compounds and carboxylic acids, followed by the probable sequestration of Al into vacuoles, may represent a more effective tolerance mechanism in bryophytes. In our study, Al exposure induced accumulation of total phenolics and flavonoids in *H. cupressiforme* and *P. euchloron*, accompanied by increased PAL activity, paralleling responses typically observed in seed plants. In contrast, phenolic levels decreased in the young shoots of *B. unguiculata*, suggesting that bryophyte species differ in their capacity to use phenolic compounds for Al detoxification. Regarding the phenylpropanoid profile, comparative studies of angiosperms and bryophytes (liverworts and mosses) indicate that although many phenylpropanoid compounds common in angiosperms are absent from bryophytes, coumarins, flavones, and flavonols are shared between angiosperms and true mosses [35]. Phenolics and flavonoids also possess ROS-scavenging properties [36,37], and the reduced PPO activity observed in all three species could further enhance their antioxidant potential, as oxidation by PPO diminishes the antioxidant effectiveness of phenolic compounds.

### 3.5. Carboxylates in Species-Specific Metabolic Adjustment and Al Detoxification

Carboxylic acids contribute not only to external Al^3+^ detoxification in higher plants but also play a prominent role in internal Al^3+^ chelation [33]. Among these, malate and citrate are ubiquitous as core tricarboxylic acid-cycle (TCA) intermediates that occur in nearly all species, whereas high oxalate accumulation is restricted to certain plant families. Tartaric acid exhibits an even more limited distribution, with substantial levels reported in only a few taxa [38]. The strongest Al-chelating ability is reported for citrate, followed by oxalate and malate [39]. Although no evidence supports a direct role of intracellular tartaric acid in Al detoxification in higher plants, its capacity to chelate metals in soils has been described [40].

In our study, four carboxylates with established (oxalic, malic, and citric) or potential (tartaric) Al^3+^-chelating capacity were detected in bryophyte leafy shoots. Their baseline concentrations showed no major interspecific differences, but the Al-induced changes in their profiles varied markedly, suggesting divergent detoxification strategies. *B. unguiculata* responded to Al with a broad increase in all four acids in both young and old shoots, consistent with internal chelation and vacuolar sequestration mechanisms known from seed plants. *P. euchloron* showed a similar increase, primarily in young shoots, whereas *H. cupressiforme* displayed reductions in both developmental stages. These patterns indicate clear species-specific differences in the deployment of carboxylic acids for internal detoxification among bryophytes.

Reports on primary metabolites, including carboxylic acids, in bryophytes are scarce, with most studies focusing on secondary metabolites [41]. Some evidence exists for malate accumulation in response to Cd stress in *Tortella tortuosa* [42]; however, to our knowledge, no data are available on citrate, oxalate, or tartrate accumulation under either stress or non-stress conditions. In another group of non-seed plants, pteridophytes, tartaric acid is known to occur as caffeoyl-tartaric acid esters [43]. In higher plant Al accumulators such as buckwheat (*Fagopyrum esculentum* Moench.), hydrangea (*Hydrangea macrophylla* (Thunb.) Ser.), and tea (*Camellia sinensis* (L.) Kuntze), oxalate is the dominant Al-chelating carboxylate, followed by citrate [44,45,46].

In addition to the above-mentioned C3 carboxylates with well-established roles in metal chelation, our analysis of C2 carboxylates, e.g., glyoxylic and glycolic acids, revealed distinct Al-induced metabolic responses. Enhanced glyoxylate- and glycolate-related metabolism has previously been linked to stress responses in bryophytes, including exposure to Cd [42]. In higher plants, the glyoxylate cycle functions as a shortcut in the TCA cycle that avoids carbon loss and enables the conversion of acetyl CoA into succinate and malate, which are produced directly without CO_2_ release and used as precursors for gluconeogenesis and glucose synthesis, particularly during seed germination. It is neither general nor constitutive but rather highly specific to particular tissues and developmental stages [47]. The pronounced interspecific differences observed in this study, together with the age-dependent responses within species, underscore divergent metabolic adjustments to Al stress among bryophytes and highlight the importance of developmental stage, warranting further investigation.

Under conditions of metabolic stress, the decarboxylation of glyoxylate to formate could represent an auxiliary detoxification and regulatory mechanism. In our work, formic acid accumulated consistently in both young and old shoots of all three species. Although formic acid is known to accumulate in certain higher plants, such as *Urtica dioica* L. [48], it has not been reported in metabolomic studies of bryophytes [32,35,41,42], despite its occurrence in lichens [49]. Notably, its basal concentration in our study was substantially higher than that of oxalate, malate, and citrate. Glyoxylate decarboxylation to formate in plants appears to function as a regulatory route that limits glyoxylate accumulation and links photorespiratory metabolism with C1 pathways and respiration under conditions of stress [50]. However, the role of formic acid in Al tolerance remains unclear, as its chemical properties suggest only a limited chelating capacity relative to these other carboxylic acids.

### 3.6. Callose Deposition and Its Limited Role in Al Tolerance

Callose (β 1,3 glucan), which is typically deposited around plasmodesmata, has also been implicated in stress responses [51]. In higher plants, callose accumulation is an indicator of Al stress [52]. It has been proposed to function as a physical barrier that protects the plasma membrane and restricts metal entry into the protoplast [53]. In this study, however, callose content remained unchanged or even decreased significantly in *P. euchloron* under Al exposure. Moreover, the basal callose concentration (~20–30 µg g^−1^ FW) was approximately one order of magnitude lower than values typically reported for higher plants (~200–800 µg g^−1^ FW) [54,55]. These findings indicate that in the studied bryophytes, enhanced callose deposition is not a principal mechanism for limiting Al entry.

### 3.7. Evidence for Al Immobilization and Cell-Wall-Based Al Tolerance

The cell wall is the primary interface for interactions with metal ions, as its negatively charged functional groups readily bind Al^3+^ [56]. Among its components, polysaccharides, particularly pectins and hemicelluloses, play central roles in Al binding due to their abundance of carboxyl groups [57]. In this study, microscopy and FTIR analysis provide strong evidence that Al is predominantly associated with the cell wall. Morin staining revealed substantial Al localization in cell walls, especially in *H. cupressiforme*. Consistently, FTIR spectra showed enhanced absorption in regions corresponding to uronic acids and pectins following Al exposure, indicating that cell-wall polysaccharides serve as major Al-binding ligands.

In higher plants, increased pectin and hemicellulose contents in roots are often associated with greater Al retention and consequently higher susceptibility to Al-induced rhizotoxicity [58]. Information on leaf responses in higher plants, however, remains limited. In contrast, the enhanced cell-wall polysaccharide-mediated Al binding observed in bryophyte leafy shoots in this study was not associated with increased toxicity; rather, it may represent an effective immobilization of Al within the apoplast, thereby reducing cytoplasmic exposure.

Structural modifications of the cell wall under metal stress have also been reported in bryophytes. For example, cell-wall thickening has been observed under Pb exposure [59], and relationships between cell-wall thickness and tolerance to Fe and Zn toxicity have also been documented [60]. In our study, the observed interspecific differences suggest that bryophytes vary substantially in their capacity to modify cell-wall chemistry in response to Al exposure, as discussed below.

### 3.8. Interspecific Differences in Al Accumulation and the Expression of Al Detoxification Mechanisms

Morin staining of laboratory-acclimated mosses cultivated for 12 weeks under Al treatment revealed clear interspecific differences in Al accumulation. The strongest fluorescence signal was observed in *P. euchloron*, followed by *H. cupressiforme*, whereas *B. unguiculata* exhibited the weakest staining (Figure 3). The lower Al accumulation in *B. unguiculata* relative to the other two species was confirmed by chemical analysis of the leafy shoots (Table 1). Although direct comparisons between field-collected and experimentally treated specimens should be made with caution because of variations in substrate characteristics and environmental conditions, a similar pattern was observed under natural conditions. Among the three species, *P. euchloron* exhibited the highest maximum Al concentration, whereas *B. unguiculata* showed the lowest values (Table 1). The consistency of these patterns under both field and controlled conditions suggests that interspecific differences in Al accumulation are largely determined by intrinsic species traits rather than environmental variation alone.

In *P. euchloron*, high Al accumulation was associated with intense Al–morin fluorescence in the cell wall and pronounced enhancement of FTIR spectral bands corresponding to pectins and uronic acids, reflecting strong cell-wall Al binding capacity (Figure 9). Concurrently, Al exposure induced coordinated increases in phenolics, flavonoids, and carboxylic acids. This integrated response suggests that *P. euchloron* combines efficient external detoxification (cell-wall immobilization) with a well-developed internal detoxification system, representing the most comprehensive Al tolerance strategy among the studied species.

In contrast, spectral shifts revealed that *B. unguiculata* showed very weak cell wall Al–morin signals and only minor enhancement of Al-binding capacity. Although carboxylic acids increased under Al stress, no corresponding rise in phenolics or flavonoids was detected. The absence of a coordinated internal detoxification response, together with limited cell-wall-mediated immobilization, indicates that *B. unguiculata* possesses the least effective Al detoxification system and is therefore likely the most susceptible species among the three. Indeed, the inhibitory effect of Al on chlorophyll concentrations was strongest in this species, significantly affecting both chlorophyll *a* and *b*.

*H. cupressiforme* employed an intermediate but distinct strategy. Concentrations of most carboxylic acids declined under Al exposure, suggesting that internal detoxification relies mainly on phenolic compounds rather than carboxylic acids. Notably, this species displayed the strongest Al–morin fluorescence in the cell wall and the greatest increase in Al binding capacity, even exceeding that of *P. euchloron*. These findings indicate a predominant reliance on cell-wall-based detoxification mechanisms, highlighting a strategy that emphasizes immobilization over intracellular chelation.

### 3.9. Ecological Implications of Al Accumulation

The ecological significance of the exceptionally high Al accumulation observed in bryophytes remains largely unresolved. While Al is generally considered toxic, the beneficial effects of moderate Al concentrations have been reported in several vascular plants adapted to acidic environments [15,61]. Comparable evidence is currently lacking for bryophytes. In the present study, the absence of visible toxicity symptoms and the presence of efficient Al sequestration mechanisms suggest a high degree of adaptation to Al-rich environments. Moreover, the Al-treated plants developed leafy shoots more rapidly and formed denser mats than the untreated controls (Figure S2), suggesting that Al may confer physiological advantages under acidic conditions. Such adaptations may contribute to the successful colonization of acidic, Al-rich habitats by bryophytes. In addition, by accumulating and retaining large amounts of Al, bryophytes may play an important role in metal immobilization and nutrient cycling in acidic-soil ecosystems. Further studies are needed to determine whether Al accumulation provides direct physiological benefits to bryophytes or primarily reflects an exceptional capacity for Al tolerance.

## 4. Materials and Methods

### 4.1. Study Area, Collection of Specimens, and Identification

The study area is located in northern Iran (Guilan Province), in the western part of the southern Caspian region, and encompasses approximately 200 km^2^. It extends between 49°35′–49°55′ E and 37°06′–37°14′ N at elevations ranging from 0 to 111 m above sea level. According to Djamali et al. [62], the region is classified as temperate oceanic.

Bryophyte species were collected during the winters of three consecutive years (2023–2025) from two habitat types: (i) tea plantations and (ii) adjacent unmanaged areas extending toward natural Hyrcanian forest stands. These habitat types were selected to encompass the major environmental variation within the study area, particularly differences in land use and soil acidity. For each species, 4–15 specimens were collected from the same locality, depending on species abundance and availability. Sampling was conducted across the study area to maximize species diversity and to include representatives from the different microhabitats and substrates encountered.

In addition to bryophytes, a limited number of pteridophyte and lichen species were also sampled. The final dataset included 28 bryophyte species (four liverworts and 24 mosses), six pteridophyte species (five ferns and one horsetail), and four lichen species (Table S7). Soil samples were collected at depths of 0–25 cm to evaluate the plants’ physical and chemical properties (Table S5).

The collected vegetation was carefully labeled, placed in plastic bags, and transported to the laboratory. Specimens were identified using standard bryofloristic and taxonomic references [16,63,64,65]. Voucher specimens are preserved in the Herbarium of the Agricultural and Natural Resources Research Center of Guilan (GILAN).

### 4.2. Elemental Analyses of Collected Specimens

The collected specimens were first washed under running tap water for 10 min to remove dust and adhering soil particles. They were then washed with 1% (*v*/*v*) HCl (ultrapure, Merck, Darmstadt, Germany), rinsed twice with distilled water for 15 min each, given a final rinse with deionized water, and gently blotted dry with filter paper. Bryophyte specimens were either separated into shoots from the upper and lower layers of the bryophyte mat (hereafter referred to as “young” and “old” shoots, respectively) or analyzed as whole mats. For pteridophytes, available plant parts (sterile and fertile fronds, stipes, and rhizomes) were separated and analyzed individually. In total, 416 specimens were prepared and subjected to the elemental analyses described below.

Plant samples were digested using a microwave digestion system (ETHOS EASY, Milestone Srl, Sorisole, Italy) with a mixture of 3 mL of concentrated HNO_3_ and 2 mL of H_2_O_2_ for 1 h. After digestion, the solutions were transferred to 25 mL volumetric flasks and diluted to volume with deionized water. Concentrations of Al and several other elements were determined, including essential micronutrients [boron (B), copper (Cu), iron (Fe), manganese (Mn), molybdenum (Mo), nickel (Ni), and zinc (Zn)]; potentially toxic non-essential metals [cadmium (Cd), cobalt (Co), chromium (Cr), and lead (Pb)]; and essential macronutrients [calcium (Ca), potassium (K), magnesium (Mg), phosphorus (P), and sulfur (S)]. Titanium (Ti) was also included in the analysis as a potential indicator of soil contamination [66].

Measurements were performed using inductively coupled plasma optical emission spectroscopy (ICP-OES; Spectro-Genesis EOP II, Spectro Analytical Instruments GmbH, Kleve, Germany). Analytical precision and accuracy were assessed using the certified reference material GBW10015 (Spinach; Institute for Geophysical and Geochemical Exploration, Langfang, China).

### 4.3. Culture of Bryophytes Under Growth Chamber Conditions

Bryophyte mats of *Barbula unguiculata*, *Palamocladium euchloron*, and *Hypnum cupressiforme* were collected in the field, placed in plastic bags, and lightly sprayed with distilled water to maintain moisture during transport to the laboratory. Upon arrival, the mats were gently rinsed with distilled water and subdivided into smaller, uniform portions for cultivation. The samples were transferred onto thin sponge pads prewashed with distilled water and autoclaved. The sponge pads were placed in transparent polypropylene containers (20 cm × 12 cm × 10 cm; L × W × H) and loosely covered with lids to minimize evaporation while allowing gas exchange. The cultivated mosses were supplied with a low-strength Hoagland nutrient solution (pH 4.0) [67], applied directly to the sponge pads.

After a two-week acclimation period under these culture conditions, two Al treatments were applied: a control without Al (–Al) and a treatment with 150 μM Al in the form of AlCl_3_. Throughout the cultivation period, sponge pads were replaced weekly to prevent algal growth, and fresh nutrient solutions were applied.

The mosses were grown in a controlled-environment growth chamber under a 16/8 h day/night photoperiod, with temperatures of 27/16 °C (day/night), relative humidity of 70–80%, and illumination from fluorescent lamps providing a photon flux density of approximately 200 μmol m^−2^ s^−1^. After 12 weeks of exposure to the Al treatments, the mosses were harvested for physiological and biochemical analyses.

### 4.4. Quantification of Al, Pigments, Carbohydrates, and Proline in Laboratory-Cultivated Mosses

Oven-dried samples were wet-digested in a sulfuric acid–H_2_O_2_ mixture for 2–3 h at 150–250 °C. Following digestion, the residue was dissolved in HCl and diluted to a known volume with distilled water. Aluminum concentrations were determined by inductively coupled plasma atomic emission spectroscopy (ICP-AES; ICP5000DV, PG Instruments Ltd., Leicestershire, United Kingdom).

Leaf chlorophyll and carotenoids were extracted in cold 80% acetone in darkness at 4 °C. After measuring absorbance at 646, 663, and 470 nm, pigment concentrations were calculated on a fresh-weight basis [68]. For non-structural carbohydrate analysis, fresh tissues were homogenized in 96% ethanol at 4 °C. After centrifugation, soluble sugars in the supernatant were quantified using the anthrone–sulfuric acid method, with glucose as a standard. The pellet was extracted with 80% dimethyl sulfoxide (DMSO) (ultrapure, Merck, Darmstadt, Germany) containing 8 M HCl (8:2, *v*/*v*) and incubated at 60 °C for 30 min to solubilize starch. Following centrifugation, starch was measured using Lugol’s reagent (2% KI, 0.2% I_2_), and absorbance was recorded at 620 nm. Starch concentration was calculated from a maize starch standard curve [69]. Proline concentration was determined according to Bates et al. [70]. Fresh tissue was extracted with 3% (*w*/*v*) sulfosalicylic acid (ultrapure, Merck, Darmstadt, Germany), and the extract was reacted with acid ninhydrin (ultrapure, Merck, Darmstadt, Germany) and glacial acetic acid (Neutron Pharmachemical Co., Tehran, Iran) at 100 °C for 1 h. After cooling, the chromophore was extracted with toluene, and absorbance was measured at 520 nm. Proline concentration was calculated using an L-proline standard curve. Callose content was determined using aniline blue staining and quantified with a spectrofluorometer (Shimadzu RF-5301PC, Shimadzu, Kyoto, Japan) at excitation and emission wavelengths of 420 and 530 nm, respectively [55].

### 4.5. Histochemical Analyses of Laboratory-Cultivated Mosses

Whole leafy shoots approximately 1.0 cm in length were used for histochemical staining. For hematoxylin staining, the shoots were first rinsed with distilled water for 15 min at 25 °C and then incubated in microtubes containing 0.2% hematoxylin (Merck, Darmstadt, Germany) and 0.02% (*w*/*v*) potassium iodide (KI) for 20 min. After staining, the shoots were washed three times with distilled water, mounted on glass slides, and photographed [71].

For the detection of Al using morin, the shoots were rinsed with distilled water and then incubated in 100 μM of morin (Fluka Chemie AG, Buchs, Switzerland) prepared in 10 mM MES buffer (pH 5.5) for 30 min. Subsequently, the samples were washed twice with MES buffer and once with deionized water for 5 min each [72]. To observe the fluorescence emitted from the morin–Al complex, stained shoots were mounted in distilled water on glass slides and examined using a fluorescence microscope (OPTIKA B-500TiFL, Optika S.r.l., Ponteranica, Italy) equipped with an excitation B filter (EX 450–490 nm, DM 495 nm, EM 500–550 nm). Images were acquired with identical exposure settings for all species and for both the control and Al-treated samples, using a microscope-mounted digital camera (OPTIKAM PRO COOL 5, 4083.CL5, Optika S.r.l., Ponteranica, Italy).

H_2_O_2_ accumulation was visualized using 1% DAB (3,3′-diaminobenzidine). Staining was carried out under light at 25 °C for 8 h. The samples were then washed with distilled water and cleared in boiling 95% ethanol, which enabled visualization of the deep brown polymerization product. The leafy shoots were photographed immediately after clearing [73].

Loss of plasma membrane integrity was assessed by staining with Evans blue (Merck, Darmstadt, Germany). Leafy shoots were immersed in 10 mL of Evans blue solution (0.025%, *w*/*v*, in 100 μM CaCl_2_, pH 5.6) for 20 min. After washing with distilled water, the stained shoots were photographed [73].

### 4.6. Activity of Antioxidant Enzymes and Concentration of Oxidant Metabolites

Total superoxide dismutase (SOD, EC 1.15.1.1) activity was determined based on the inhibition of NBT (p-nitro blue tetrazolium chloride) reduction and the consequent decrease in blue formazan formation under light [55]. Catalase (CAT, EC 1.11.1.6) activity was assayed by monitoring the decrease in the absorbance of H_2_O_2_ at 240 nm. The enzyme activity was calculated using an extinction coefficient for H_2_O_2_ of 0.041 mM^−1^ cm^−1^ and expressed as µmol H_2_O_2_ mg^−1^ protein min^−1^ [55]. Ascorbate peroxidase (APX, EC 1.11.1.11) activity was assayed by monitoring the oxidation of ascorbic acid as a decrease in absorbance at 290 nm. Enzyme activity was calculated using an extinction coefficient of 2.8 mM^−1^ cm^−1^ for ascorbic acid and expressed as µmol ascorbate oxidized mg^−1^ protein min^−1^ [66]. Peroxidase (POD, EC 1.11.1.7) was extracted using 10 mM phosphate buffer (pH 7.0) and measured at 470 nm with guaiacol (Merck, Darmstadt, Germany) as the substrate; enzyme activity was calculated using an extinction coefficient of 26.6 mM^−1^ cm^−1^ for tetraguaiacol [66].

H_2_O_2_ concentration was determined in extracts prepared with 0.1% (*w*/*v*) trichloroacetic acid using 0.5 mM potassium iodide (KI), and absorbance was recorded at 390 nm [74]. Malondialdehyde (MDA) content was measured spectrophotometrically at 532 nm after reacting with thiobarbituric acid and quantified using a calibration curve prepared with 1,1,3,3-tetraethoxypropane (Sigma-Aldrich, St. Louis, MO, USA) [75].

### 4.7. Activity of Phenolic-Metabolizing Enzymes and Concentration of Phenolic Compounds

Phenylalanine ammonia-lyase (PAL, EC 4.3.1.5) activity was quantified as the amount of trans-cinnamic acid produced from L-phenylalanine during 30 min of incubation at 30 °C. Absorbance was recorded at 290 nm, and concentrations were calculated using an extinction coefficient of 9.63 mM^−1^ cm^−1^ [76]. Polyphenol oxidase (PPO, EC 1.14.18.1) activity was assessed by measuring the increase in absorbance at 334 nm due to pyrogallol oxidation [77]. Total soluble protein content was determined using Bradford reagent with bovine serum albumin (Merck, Darmstadt, Germany) as the standard.

Soluble phenolics were extracted three times in 70% aqueous methanol at 4 °C in darkness. After centrifugation, the combined supernatants were analyzed using Folin–Ciocalteu reagent (Sigma-Aldrich, St. Louis, MO, USA), with gallic acid used as the calibration standard [78]. Total flavonoids were extracted in 95% ethanol and quantified at 415 nm using the AlCl_3_ colorimetric method. A calibration curve based on quercetin (Sigma-Aldrich, St. Louis, MO, USA) was used, and flavonoid concentrations were expressed as quercetin equivalents [79].

### 4.8. Analysis of Carboxylic Acids

For the analysis of carboxylic acids (tartaric, malic, citric, and oxalic acids, along with formic, glyoxylic, and glycolic acids), freeze-dried samples were weighed and extracted in 5% H_3_PO_4_, followed by centrifugation at 10,000 *g* for 20 min. Approximately 700 μL of the supernatant was mixed with a silica-based cation exchanger (LiChrolut SCX, Merck, Darmstadt, Germany) at a ratio of 100 mg of sorbent per 1 mL of extract. After 10–15 min, the samples were centrifuged again at 10,000 *g* for 10 min, and the resulting supernatant was used for HPLC analysis of carboxylic acids using a Shimadzu LC-10AT system (Shimadzu, Kyoto, Japan). Separation was achieved with 18 mM KH_2_PO_4_ (pH 2.1) as the mobile phase at a flow rate of 0.5 mL min^−1^, a column temperature of 28 °C, and UV detection at 215 nm [80].

### 4.9. FTIR Analysis

Fourier transform infrared (FTIR) spectroscopy was conducted on a Bruker Tensor 27 FT-IR Spectrum System (Tensor 27, Bruker Optik GmbH, Ettlingen, Germany) in the mid-IR region (400–4000 cm^−1^), with the instrument operated in absorption mode at a resolution of 1.0 cm^−1^. Subsamples of the freeze-dried leafy shoot samples, each consisting of a pooled mixture of four biological replicates, were ground to a fine powder, mixed with dried potassium bromide (KBr) (Merck, Darmstadt, Germany), pressed into pellets, and used for analysis. Spectra in the range of 900–1800 cm^−1^, which corresponded to pectin and uronic acids [81,82], were plotted for each sample.

### 4.10. Statistical Analyses

For each collected species, 4–12 specimens from the same location and year were analyzed by ICP-OES. To summarize Al accumulation across species, the minimum and maximum concentrations recorded for each species were reported, irrespective of sampling time, location, or plant part (Table 1). Pearson’s correlation coefficients among elemental concentrations were calculated using Minitab 18 (Minitab, LLC, State College, PA, USA).

*Palamocladium euchloron* and *Amblystegium serpens* were selected for detailed statistical analyses because of their high frequency of occurrence and the availability of complete datasets encompassing different collection sites, substrate types, and shoot-age categories. In *P. euchloron*, the effects of shoot age (young versus old shoots) and collection site (tea plantation versus forest stand) on Al accumulation were tested separately using one-way ANOVA. The influence of substrate type on Al concentration was evaluated for both *P. euchloron* and *A. serpens* by one-way ANOVA (Table S1). In ferns, differences in Al concentration between sterile and fertile fronds, representing younger and older tissues, respectively, were likewise assessed using one-way ANOVA (Table S3).

Growth-chamber experiments were conducted in a completely randomized design with four independent containers per treatment (*n* = 4). Mean comparisons were performed using Tukey’s test at *p* < 0.05 with SigmaStat 3.02 (Systat Software Inc., San Jose, CA, USA). Graphs were prepared using GraphPad Prism 9.0 (GraphPad Software LLC, San Diego, CA, USA).

## 5. Conclusions

Bryophytes are key components of acidic habitats, yet their interactions with Al remain poorly understood. This study demonstrates that they can accumulate substantial amounts of Al, primarily localized in leafy shoots within cell walls, indicating that apoplastic binding is a major tolerance mechanism. In addition, adjustments in phenolic metabolism, antioxidant activity, and carboxylic acid profiles contribute to Al tolerance, with marked variability among species.

These findings highlight the ecological significance of bryophytes and their active role in Al cycling in acidic environments. By revealing multiple physiological and biochemical strategies for coping with Al, this work provides a foundation for future research on the ecological and evolutionary dimensions of metal tolerance in early land plants. Further integrative studies combining field, experimental, and molecular approaches will be essential in clarifying how bryophytes regulate Al uptake, immobilization, and detoxification across diverse habitats.

## Figures and Tables

**Table 1:** Non-seed plant and lichen species collected from acidic soils in tea gardens and the Hyrcanian forests of northern Iran and their ranges of aluminum (Al) concentration.

Species	Al (µg g^−1^ DW)
**Bryophytes**	
*Amblystegium serpens* (Hedw.) Schimp.	1039–7000
*Atrichum undulatum* (Hedw.) P. Beauv.	7545–13,950
*Barbula unguiculata* Hedw.	1990–3139
*Brachythecium rutabulum* (Hedw.) Schimp.	1039–20,720
*Bryum capillare* Hedw.	5432–6979
*Bryum argenteum* Hedw.	5200–5695
*Cryphaea heteromalla* (Hedw.) D. Mohr	2170–3223
*Ctenidium molluscum* (Hedw.) Mitt.	2336–6376
*Dicranum* sp.	3009–24,034
*Fissidens taxifolius* Hedw.	6389–8956
*Fossombronia caespitiformis* De Not. ex Rabenh.	11,844–28,422
*Frullania tamarisci* (L.) Dumort.	3268–3593
*Hypnum cupressiforme* Hedw.	2978–3499
*Lunularia cruciata* (L.) Lindb.	4718–26,112
*Neckera besseri* Lobarz.	2415–3420
*Neckera complanata* (Hedw.) Huebener	1330–2091
*Neckera crispa* Hedw.	1114–5149
*Oxyrrhynchium hians* (Hedw.) Loeske	1720–2079
*Palamocladium euchloron* (Bruch ex Müll. Hal.) Wijk & Margad.	1297–6549
*Physcomitrium pyriforme* (Hedw.) Brid.	2780–4156
*Plagiomnium rostratum* (Schard.) T. J. Kop	1364–7452
*Plagiomnium undulatum* (Hedw.) T. J. Kop.	4939–7795
*Radula complanata* (L.) Dumort.	4678–12,353
*Schistidium apocarpum* (Hedw.) Bruch & Schimp	5802–6093
*Sciuro-hypnum oedipodium* (Mitt.) Ignatov & Huttunen	1301–15,620
*Thamnium alopecurum* (Hedw.) Schimp.	1876–13,420
*Thuidium delicatulum* (Hedw.) Schimp.	9871–11,310
*Tortella tortuosa* (Hedw.) Limpr.	1990–3139
**Pteridophytes**	
*Asplenium adiantum-nigrum* L.	174–1091
*Asplenium scolopendrium* L.	131–521
*Dryopteris raddeana* (Fomin) Fomin	233–936
*Equisetum telmateia* Ehrh.	489–1345
*Polypodium vulgare* L.	97–367
*Pteris cretica* L.	138–813
**Lichens**	
*Flavoparmelia caperata* (L.) Hale	2758–3541
*Lepraria leuckertiana* (Zedda) L. Saag	2816–9154
*Punctelia subrudecta* (Nyl.) Krog	1063–2138
*Xanthoria parietina* (L.) Th. Fr.	4228–4885

**Table 2:** Concentrations of Al (µg g^−1^ DW), chlorophylls (Chl *a*, *b*) (mg g^−1^ FW), carotenoids (µg g^−1^ FW), soluble sugars and starch (mg g^−1^ FW), proline (µmol g^−1^ FW), and callose (µg g^−1^ FW) in leafy shoots of three bryophyte species cultivated without Al (–Al) or with 150 µM Al (+Al, as AlCl_3_) at pH 4.0 for 12 weeks under controlled environmental conditions. Values for each species followed by the same letter are not significantly different at *p* < 0.05.

	**Al**	**Chl *a***	**Chl *b***	**Carotenoids**
	*Barbula unguiculata*			
–Al	304 ± 88 ^b^	0.84 ± 0.02 ^a^	0.23 ± 0.02 ^a^	57.6 ± 5.58 ^a^
+Al	1129 ± 100 ^a^	0.59 ± 0.01 ^b^	0.08 ± 0.02 ^b^	6.4 ± 0.71 ^b^
	*Palamocladium euchloron*			
–Al	195 ± 33 ^b^	0.81 ± 0.05 ^a^	0.25 ± 0.01 ^a^	58.2 ± 3.54 ^a^
+Al	1929 ± 260 ^a^	0.48 ± 0.01 ^b^	0.26 ± 0.01 ^a^	5.2 ± 1.64 ^b^
	*Hypnum cupressiforme*			
–Al	340 ± 71 ^b^	0.75 ± 0.03 ^a^	0.29 ± 0.01 ^a^	37.9 ± 8.25 ^a^
+Al	1676 ± 252 ^a^	0.70 ± 0.03 ^a^	0.17 ± 0.01 ^b^	3.5 ± 1.26 ^b^
	**Soluble sugars**	**Starch**	**Proline**	**Callose**
	*Barbula unguiculata*			
–Al	33.9 ± 1.44 ^b^	0.24 ± 0.01 ^a^	25.5 ± 4.65 ^a^	32.1 ± 4.9 ^a^
+Al	48.2 ± 3.21 ^a^	0.21 ± 0.03 ^a^	11.8 ± 0.57 ^b^	28.1 ± 1.9 ^a^
	*Palamocladium euchloron*			
–Al	39.9 ± 1.37 ^b^	0.55 ± 0.04 ^a^	10.8 ± 2.40 ^a^	31.7 ± 4.6 ^a^
+Al	56.5 ± 8.00 ^a^	0.40 ± 0.05 ^b^	5.44 ± 0.91 ^b^	25.4 ± 1.5 ^b^
	*Hypnum cupressiforme*			
–Al	15.6 ± 3.06 ^b^	0.26 ± 0.05 ^b^	5.93 ± 0.43 ^a^	27.6 ± 3.5 ^a^
+Al	30.5 ± 2.31 ^a^	0.40 ± 0.03 ^a^	5.36 ± 0.99 ^a^	25.9 ± 1.0 ^a^

## Data Availability

The data presented in this study are available on request from the corresponding author.

## Supplementary Materials

The following supporting information can be downloaded at https://www.mdpi.com/article/10.3390/plants15121877/s1, Table S1. Results of one-way ANOVA assessing the effect of shoot age and collection site in *P. euchloron* and the effect of substrate type on Al accumulation in *P. euchloron* and *A. serpens*. Reported statistics include degrees of freedom (df), mean square (MS), F value (F), and probability level (*p*). Table S2. Pteridophyte species collected from acidic soils in tea gardens and Hyrcanian forests of northern Iran and ranges of Al concentrations in different plant fractions. Table S3. Results of one-way ANOVA evaluating the effect of frond age and/or fertility status on Al accumulation in different fern species. Reported statistics include degrees of freedom (df), mean square (MS), F value (F), and probability level (*p*). Table S4. Concentrations (µmol g^−1^ DW) of glyoxylic, glycolic, and formic acids in young and old leafy shoots of three bryophyte species cultivated without Al (–Al) or with 150 µM Al (+Al, as AlCl_3_) at pH 4.0 for 12 weeks under controlled environmental conditions. Values followed by the same letter are not significantly different at *p* < 0.05. Table S5. Physical and chemical properties and available or total concentrations of selected mineral elements in soil samples collected from two main locations in the study area. n.d.: not detectable. Table S6. Activities of superoxide dismutase (SOD), catalase (CAT), ascorbate peroxidase (APX), and peroxidase (POD) in leafy shoots of three bryophyte species cultivated without Al (–Al) or with 150 µM Al (+Al, as AlCl_3_) at pH 4.0 for four weeks under controlled environmental conditions. Bars indicated by the same letter are not significantly different at *p* < 0.05. Table S7. Plant species collected and analyzed from acidic soils in northern Iran, including 28 bryophytes (four liverworts and 24 mosses), six pteridophytes (five ferns and one horsetail), and four lichen species. Figure S1. Concentrations (µg g^−1^ DW) of macronutrients (Ca, K, Mg, P, S) and heavy metals (Cd, Co, Cr, Ni, Pb) in leafy shoots of bryophyte species collected from acidic soils in tea gardens and Hyrcanian forests of northern Iran. Figure S2. Representative photographs of *Barbula unguiculata* collected from the field, acclimated to laboratory conditions, and cultivated for 12 weeks in the absence (−Al) or presence (+Al, 150 µM) of aluminum. Al-treated plants exhibited more extensive leafy shoot development and denser mat formation than control plants.
