# Supplementary material for: Bryophytes as Strong Aluminum Accumulators in Acidic Soils: Cell-Wall Binding and Physiological Tolerance Mechanisms
Source: Plants (Basel). 2026 Jun 17;15(12):1877. doi: 10.3390/plants15121877 (PMC13307116; doi:10.3390/plants15121877)
Supplement: Supplementary file 1 [file plants-15-01877-s001.zip › Suppl Figure.pdf]

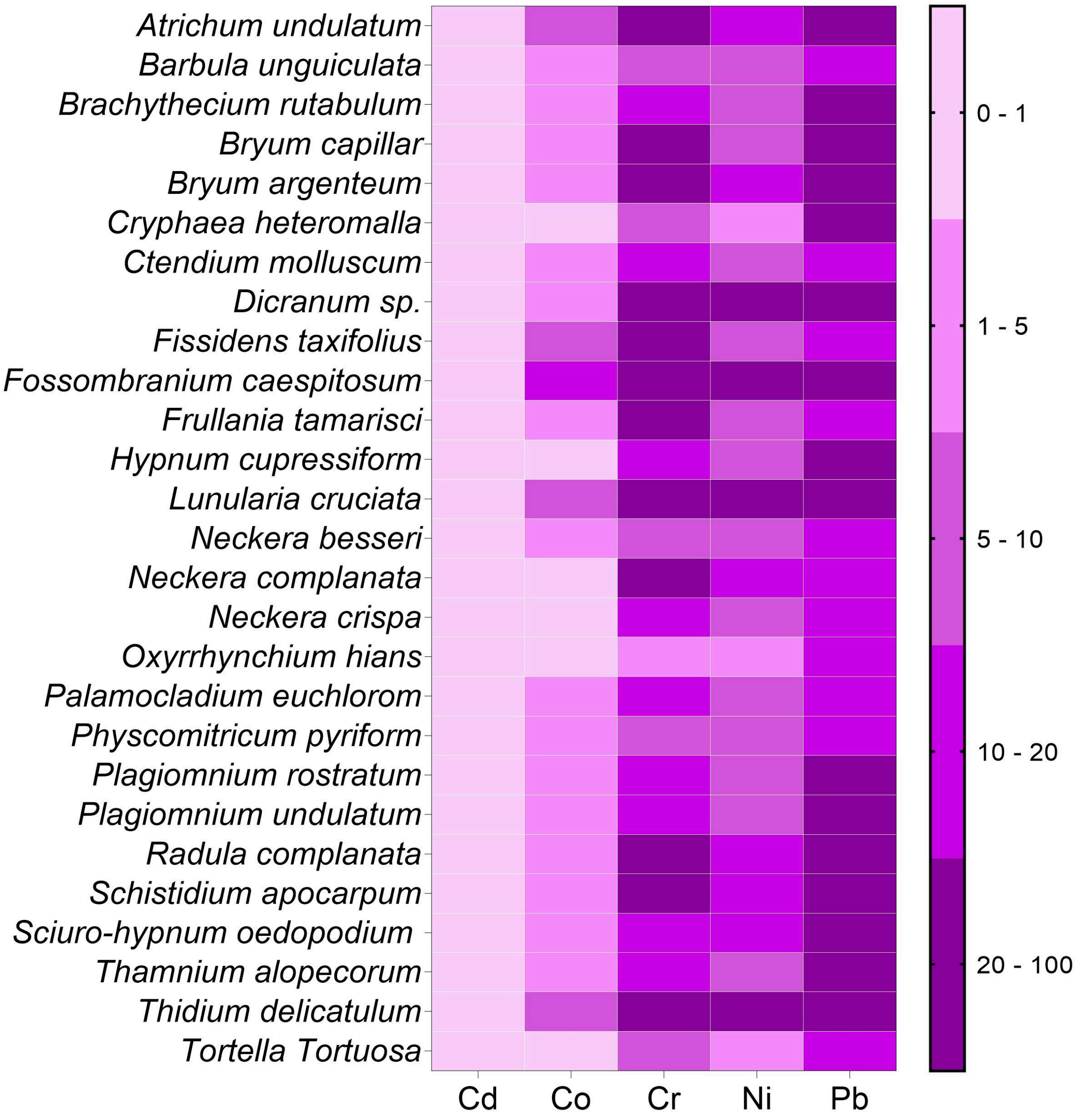

Figure S1. Concentrations (μg g^{-1} DW) of macronutrients (Ca, K, Mg, P, S) and heavy metals (Cd, Co, Cr, Ni, Pb) in leafy shoots of bryophyte species collected from acidic soils in tea gardens and Hyrcanian forests of northern Iran.

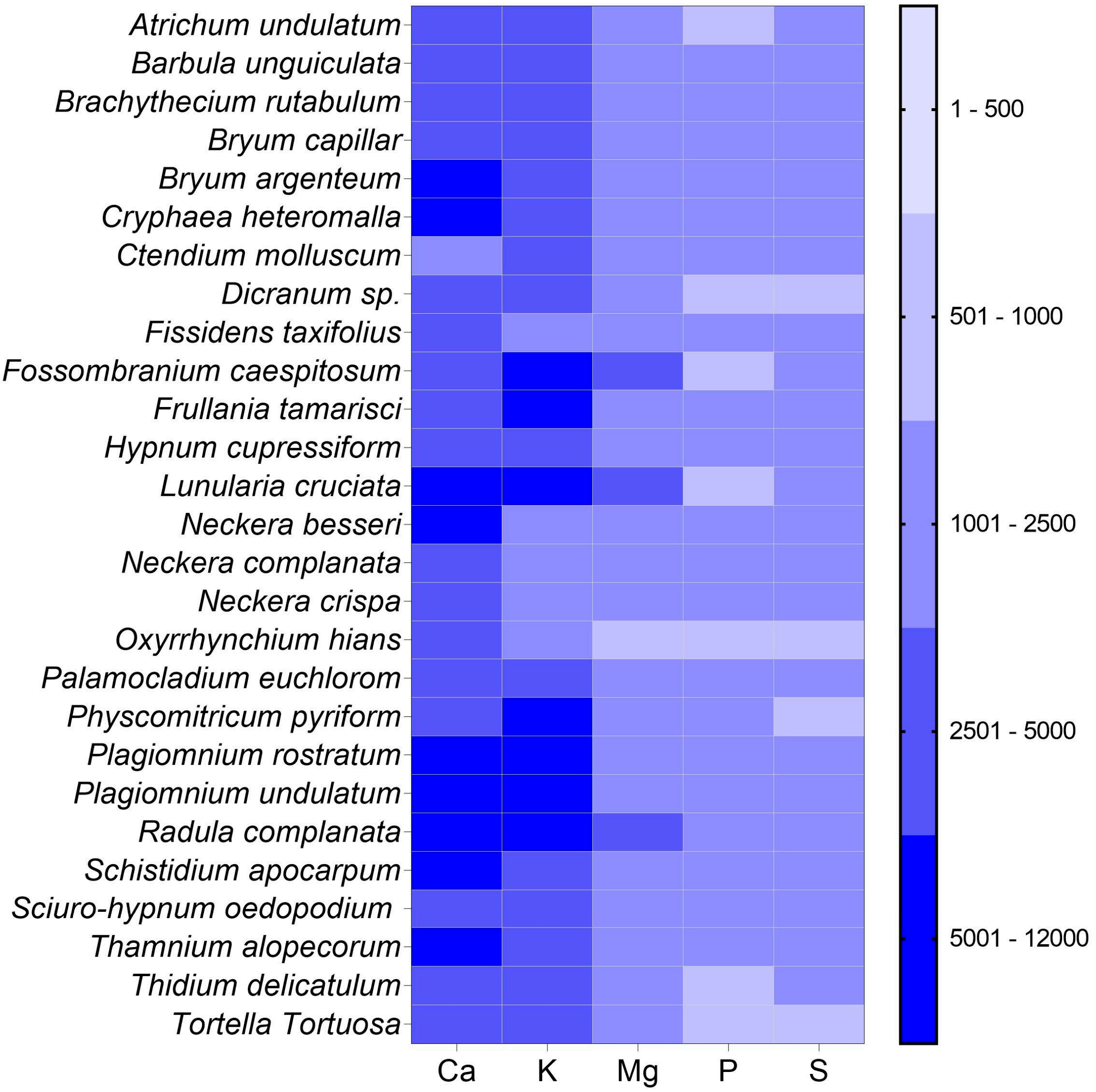

Figure S2. Representative photographs of *Barbula unguiculata* collected from the field, acclimated to laboratory conditions, and cultivated for 12 weeks in the absence (−Al) or presence (+Al, 150 μM) of aluminum. Al-treated plants exhibited more extensive leafy shoot development and denser mat formation than control plants.
