# Supplementary material for: Bryophytes as Strong Aluminum Accumulators in Acidic Soils: Cell-Wall Binding and Physiological Tolerance Mechanisms
Source: Plants (Basel). 2026 Jun 17;15(12):1877. doi: 10.3390/plants15121877 (PMC13307116; doi:10.3390/plants15121877)
Supplement: Supplementary file 1 [file plants-15-01877-s001.zip › Suppl Table.pdf]

Table S1. Physical and chemical properties and available or total concentrations of selected mineral elements in soil samples collected from two main locations in the study area.

	pH	EC (dS m^{-1})	OC (%)	Available (mg kg^{-1})						Total (mg kg^{-1})
				Al	Cu	Fe	Mn	Mo	Zn	Ti
Tea gardens	3.7	0.32	3.2	16.5	1.24	42.7	122	0.01	1.3	891
Forest	5.6	0.19	2.1	3.7	0.71	9.1	28	0.01	0.65	1125
	Total macroelements (%)					Total heavy metals (mg kg^{-1})				
	Ca	Mg	K	P	S	Cd	Co	Cr	Ni	Pb
Tea gardens	0.15	0.53	0.62	88.6	32.7	n.d.	17.5	72.9	38.6	70.4
Forest	0.28	0.73	0.93	69.8	26.1	n.d.	16.8	51.8	25.8	58.6

Table S2. Plant species collected and analyzed from acidic soils in northern Iran, including 27 bryophytes (four liverworts and 23 mosses), six pteridophytes (five ferns and one horsetail), and four lichen species.

**Species**	**Family**	**Morphological habits**
**Liverworts**		
Fossombronia caespitiformis De Not. ex Rabenh.	Codoniaceae	Thalloid liverwort
Frullania tamarisci (L.) Dumort.	Frullaniaceae	Leafy liverwort
Lunularia cruciata (L.) Lindb.	Lunulariaceae	Thalloid liverwort
Radula complanata (L.) Dumort.	Radulaceae	Leafy liverwort
**True mosses**		
Atrichum undulatum (Hedw.) P.Beauv.	Polytrichaceae	Acrocarpous moss
Barbula unguiculata Hedw.	Pottiaceae	Acrocarpous moss
Brachythecium rutabulum (Hedw.) Schimp.	Brachytheciaceae	Pleurocarpous moss
Bryum capillare Hedw.	Bryaceae	Acrocarpous moss
Bryum argenteum Hedw.	Bryaceae	Acrocarpous moss
Cryphaea heteromalla (Hedw.) D.Mohr	Cryphaceae	Pleurocarpous moss
Ctendium molluscum (Hedw.) Mitt.	Hypneaceae	Pleurocarpous moss
Dicranum sp.	Dicranaceae	Acrocarpous moss
Fissidens taxifolius Hedw.	Fissidentaceae	Acrocarpous moss
Hypnum cupressiform Hedw.	Hypneaceae	Pleurocarpous moss
Neckera besseri Lobarz.	Neckeraceae	Pleurocarpous moss
Neckera complanata (Hedw.) Huebener	Neckeraceae	Pleurocarpous moss
Neckera crispa Hedw.	Neckeraceae	Pleurocarpous moss
Oxyrrhynchium hians (Hedw.) Loeske	Brachytheciaceae	Pleurocarpous moss
Palamocladium euchlorom (Mull. Hal.) Wijk & Margad.	Brachytheciaceae	Pleurocarpous moss
Physcomitricum pyriform (Hedw.) Hamp	Funariaceae	Acrocarpous moss
Plagiomnium rostratum (Schard.) T.J.Kop.	Mniaceae	Acrocarpous moss
Plagiomnium undulatum (Hedw.) T.J.Kop.	Mniaceae	Acrocarpous moss
Schistidium apocarpum (Hedw.) Bruch & Schimp	Grimmiaceae	Acrocarpous moss
Sciuro-hypnum oedopodium (Mitt.) Ignatov & Huttunen	Brachytheciaceae	Pleurocarpous moss
Thamnium alopecorum (Hedw.) Schimp.	Thamniaceae	Pleurocarpous moss with a tree-like branching
Thidium delicatulum (Hedw.) Schimp.	Thuidiaceae	Pleurocarpous perennial moss form
Tortella Tortuosa (Hedw.) Limpr.	Pottiaceae	Acrocarpous moss
**Ferns**		
Asplenium adiantum-nigrum L.	Polypodiaceae	Herbaceous
Asplenium scolopendrium L.	Polypodiaceae	Herbaceous
Dryopteris raddeana (Fomin) Fomin	Dryopteridaceae	Herbaceous
Polypodium vulgare L.	Polypodiaceae	Herbaceous
Pteris cretica L.	Pteridaceae	Herbaceous
**Horsetails**		
Equisetum telmateia Ehrh.	Equisetaceae	Herbaceous
**Lichens**		
Flavoparmelia caperata (L.) Hale	Parmeliaceae	Foliose
Lepraria leuckertiana ***(Zedda) L. Saag***	Stereocaulaceae	Leprose
Punctelia subrudecta (Nyl.) Krog	Parmeliaceae	Foliose
Xanthoria parietina (L.) Th. Fr.	Teloschistaceae	Foliose

Table S3. Results of one-way ANOVA assessing the effect of shoot age and two-way ANOVA evaluating the effects of collection site and substrate type on Al accumulation in *Palamocladium euchloron*. Reported statistics include degrees of freedom (df), mean square (MS), F-value (F), and probability level (*P*).

Factor	Shoot age			
	df	MS	F	P
Shoot age	1	55064488.120	24.701***	<0.001
Residual	14	31208786.317	2229199.023	
Total	15	86273274.438		
	Collection site			
	df	MS	F	P
Site	1	8353545.063	6.384	0.027
Substrate	1	5411439.063	4.135	0.065
Site × Substrate	1	114413.063	0.0874	0.773
Residual	12	15702534.250	1308544.521	
Total	15	29581931.438	1972128.763	

Table S4. Results of one-way ANOVA evaluating the effect of frond age and/or fertility status on Al accumulation in different fern species. Reported statistics include degrees of freedom (df), mean square (MS), F-value (F), and probability level (*P*).

Factor	Frond age & fertility status			
	df	MS	F	P
Age & fertility status	1	1838607/332	31/552	<0/001
Residual	43	58272/240		
Total	44			

Table S5. Pteridophyte species collected from acidic soils in tea gardens and Hyrcanian forests of northern Iran, together with the ranges of Al concentrations in different plant fractions.

<table>
<tr><th>Plant species</th><th></th><th>Al concentration (µg g^{-1} DW)</th></tr>
<tr><td colspan="3">Asplenium adiantum-nigrum L. (Polypodiaceae)</td></tr>
<tr><td>Sterile fronds (young)</td><td></td><td>174–379</td></tr>
<tr><td>Sterile fronds (old)</td><td></td><td>587–647</td></tr>
<tr><td>Fertile fronds</td><td></td><td>587–1474</td></tr>
<tr><td>Stipe</td><td></td><td>895–1091</td></tr>
<tr><td>Rhizomes</td><td></td><td>1074–1123</td></tr>
<tr><td colspan="3">Asplenium scolopendrium L. (Polypodiaceae)</td></tr>
<tr><td>Sterile fronds (young)</td><td></td><td>131–237</td></tr>
<tr><td>Sterile fronds (old)</td><td></td><td>424–521</td></tr>
<tr><td>Stipe</td><td></td><td>165–245</td></tr>
<tr><td colspan="3">Dryopteris raddeana (Fomin) Fomin (Dryopteridaceae)</td></tr>
<tr><td>Sterile fronds (young)</td><td></td><td>233–494</td></tr>
<tr><td>Sterile fronds (old)</td><td></td><td>564–881</td></tr>
<tr><td>Fertile fronds</td><td></td><td>372–936</td></tr>
<tr><td>Stipe</td><td></td><td>935–1199</td></tr>
<tr><td colspan="3">Polypodium vulgare (Polypodiaceae)</td></tr>
<tr><td>Sterile fronds</td><td rowspan="2">Epiphytic on Common hornbeam (Carpinus betulus L.)</td><td>246–253</td></tr>
<tr><td>Rhizomes</td><td>301–413</td></tr>
<tr><td>Fertile fronds</td><td rowspan="2">Epiphytic on white mulberry (Morus alba L.)</td><td>101–367</td></tr>
<tr><td>Rhizomes</td><td>40–98</td></tr>
<tr><td>Sterile fronds</td><td rowspan="3"></td><td>97–127</td></tr>
<tr><td>Fertile fronds</td><td>261–362</td></tr>
<tr><td>Rhizomes</td><td>3762–4525</td></tr>
<tr><td colspan="3">Pteris cretica L. (Pteridaceae)</td></tr>
<tr><td>Sterile fronds (young)</td><td></td><td>138–327</td></tr>
<tr><td>Sterile fronds (old)</td><td></td><td>444–515</td></tr>
<tr><td>Fertile fronds</td><td></td><td>515–813</td></tr>
<tr><td>Stipe</td><td></td><td>69–168</td></tr>
<tr><td colspan="3">Equisetum telmateia (Equisetaceae)</td></tr>
<tr><td>Leaves</td><td></td><td>489–623</td></tr>
<tr><td>Leafless stalks & reproductive structures</td><td></td><td>1262–1345</td></tr>
</table>

Table S6. Concentrations (µmol g^{-1} DW) of glyoxylic, glycolic, and formic acids in young and old leafy shoots of three bryophyte species cultivated without Al (–Al) or with 150 µM Al (+Al, as $AlCl_3$) at pH 4.0 for 12 weeks under controlled environmental conditions. Values followed by the same letter are not significantly different at $P < 0.05$.

	Glyoxylic acid		Glycolic acid		Formic acid	
	Young S	Old S	Young S	Old S	Young S	Old S
			B. unguiculata			
–Al	63±4.1 ^{b}	11±2.0 ^{a}	417±22 ^{b}	245±65 ^{b}	170±9.3 ^{b}	39±5.9 ^{b}
+Al	131±2.1 ^{a}	11±1.2 ^{a}	793±49 ^{a}	481±42 ^{a}	195±9.7 ^{a}	88±9.3 ^{a}
			P. euchloron			
–Al	43±4.4 ^{b}	66±1.7 ^{b}	551±15 ^{a}	409±48 ^{b}	244±9.2 ^{b}	215±5.5 ^{b}
+Al	220±19 ^{a}	476±14 ^{a}	481±23 ^{b}	496±27 ^{a}	268±2.9 ^{a}	306±4.3 ^{a}
			H. cupressiforme			
–Al	119±12 ^{a}	248±33 ^{a}	1329±132 ^{a}	181±7.7 ^{b}	278±34.9 ^{b}	145±19 ^{b}
+Al	117±9.1 ^{a}	95±13 ^{b}	609±173 ^{b}	485±42 ^{a}	916±61.9 ^{a}	437±48 ^{a}

Table S7. Activities of superoxide dismutase (SOD), catalase (CAT), ascorbate peroxidase (APX), and peroxidase (POD) in leafy shoots of three bryophyte species cultivated without Al (–Al) or with 150 µM Al (+Al, as $AlCl_3$) at pH 4.0 for four weeks under controlled environmental conditions. Bars indicated by the same letter are not significantly different at $P < 0.05$.

	SOD	CAT	APX	POD
		Barbula unguiculata		
–Al	486±43 ^{a}	36.1±3.82 ^{a}	0.525±0.176 ^{a}	0.224±0.048 ^{a}
+Al	369±69 ^{b}	14.6±4.00 ^{b}	0.313±0.018 ^{a}	0.129±0.045 ^{b}
		Palamocladium euchloron		
–Al	461±19 ^{a}	10.97±1.19 ^{a}	0.253±0.038 ^{a}	3.92±1.57 ^{a}
+Al	412±37 ^{a}	5.58±1.70 ^{b}	0.162±0.020 ^{b}	1.60±0.46 ^{b}
		Hypnum cupressiforme		
–Al	434±39 ^{a}	63.96±6.24 ^{a}	0.410±0.068 ^{a}	0.245±0.093 ^{a}
+Al	369±16 ^{b}	22.05±4.24 ^{b}	0.282±0.033 ^{b}	0.184±0.080 ^{a}
